# Post-Traumatic Stress Disorder and Cognitive Impairment: The Great Unknown

**DOI:** 10.1192/j.eurpsy.2025.1960

**Published:** 2025-08-26

**Authors:** S. Orrego, M. Sava, S. Rozas, D. Castro, M. Delso, M. Nadales, M. S. Manzano

**Affiliations:** 1 Psiquiatria, Hospital Universitario Infanta Leonor; 2 Psiquiatria, Hospital Universitario Jose Germain; 3 Neurologia, Hospital Universitario Infanta Leonor, Madrid, Spain

## Abstract

**Introduction:**

Accumulating evidence identifies post-traumatic stress disorder (PTSD) as a significant risk factor for the development of dementia, with affected individuals demonstrating a twofold increase in dementia risk compared to the general population. Additionally, the development of dementia in these patients may exacerbate the clinical manifestations associated with PTSD, complicating proper management and treatment.

**Objectives:**

To describe a clinical case of a patient with a history of PTSD presenting with cognitive impairment, and to provide a brief review of the literature.

**Methods:**

Case description and literature review.

**Results:**

A 65-year-old male patient, originally from Colombia, with a documented history of PTSD secondary to kidnapping and armed conflict, presented to psychiatric services in Spain for initial assessment and treatment. The family reports neglect of self-care, persistent hypervigilance, nightmares, and night screams, as well as multiple attentional failures, learning difficulties, and memory impairments. The patient was referred for a neurological consultation where the evaluation included a Mini-Mental State Examination (MMSE) scoring 16/30. Cranial MRI showed no abnormalities, and amyloid PET was negative. An FDG PET scan revealed discrete hypometabolism of the medial prefrontal cortex, which could be indicative of possible frontotemporal dementia.

**Conclusions:**

The prevalence of dementia is rising globally. PTSD has been identified as a modifiable risk factor for developing dementia. Furthermore, studies show that the relationship between these conditions is bidirectional, with late-onset PTSD also potentially developing in patients with a diagnosis of dementia. The mechanisms underlying this relationship are poorly understood. It is hypothesized that both conditions share common pathophysiological pathways. PTSD manifestations in patients with dementia are often difficult to recognize, which is believed to result in an underdiagnosis of the condition. Additionally, these clinical manifestations can be confused with neuropsychiatric symptoms associated with dementia, further complicating diagnosis. The relationship between PTSD and dementia may be modifiable if patients with PTSD are provided with appropriate diagnosis and treatment, thereby improving their quality of life and prognosis.

**Disclosure of Interest:**

None Declared

